# Supplementary material for: A Deep Learning Approach to Capture the Essence of Candida albicans Morphologies
Source: Microbiol Spectr. 2022 Aug 16;10(5):e01472-22. doi: 10.1128/spectrum.01472-22 (PMC9604015; doi:10.1128/spectrum.01472-22)
Supplement: Supplemental file 10 — Supplemental material. Download spectrum.01472-22-s0010.pdf, PDF file, 0.08 MB [file spectrum.01472-22-s0010.pdf]

Supplemental Material

The space of *C. albicans* morphologies is complex and continuous

We observed considerable cell-to-cell morphological variability across the images. Heterogeneity arises due to technical variations (e.g., light intensity, focus), natural biological programs (e.g., cell cycle affecting size/shape), and transitions between morphologies (e.g., growth of a hyphal cell from germ tube). These sources of heterogeneity complicate both the manual labeling procedure during construction of the learning set and the downstream ability of an FCOS to correctly assign morphology. Our goal here is to quantitatively explore the complexity of the Varasana compendium in an unbiased manner.

Towards this end, we developed an unsupervised approach based on a Variational AutoEncoder (VAE)[1]. A VAE is a generative artificial neural network that uses a game theoretic approach involving two players: the encoder and the decoder. Instances of each object (individual cells from our ground truth annotations) are provided to the encoder. The encoder re-represents these images in the latent (hidden) layers of its network. Typically, the latent space is much smaller than the size of the original input, forcing the encoder to build succinct models capturing the most salient features of each image. The goal of the decoder is to reconstruct the original image from only the encoder's latent representation. The encoder and decoder together are penalized according to how much the reconstructed image differs from the original. This cycle is repeated for many epochs across the training and validation sets. Note that this encoding is built in an unsupervised manner as it does not make use of the class labels (morphological assignments).

Our VAE uses several convolutional layers to encode each image in a 2D latent space across all wildtype (SC5314, SN148a) cells detected by the FCOC-classifier and β parameter that down-weights the Kullbach-Leibler component of the loss function. It was constructed using the Keras for R (version 2.4) package[2]. Briefly here, the input to the network is a 128×128 pixel image. The image is subjected to a series of convolutional layers with a 5×5 kernels, 64 filters and strides that successively transformed the representation, followed by a flattening operation that reshapes the representation into a $262{,}144$ real vector, before a final reduction to a two-dimensional latent space. Layers for z mean, the $z\ log$score and for decoding all follow standard VAE procedures (see code for details). Training used the wildtype Varasana training and validation sets in batch sizes of 100 across 20 epochs. The test set objects, which include the genetically modified *C. albicans* variants from Varasana, were not used in training. In particular, we extracted each (ground truth) bounding box across these files and reshaped the images to size 128×128. We used a β=0.4 parameter to down-weight the Kullbach-Leibler portion of the loss function[3]. It is straightforward to visualize the resultant two-dimensional latent space with a scatterplot.

After training, the resultant encoder was applied to all cells from the test and validation sets and the two-dimensional latent space visualised (**Supplemental Figure 8**). Rather than islands of distinct isolated cells, we observe an unbroken continuum in both the V1 and V2 axes. Although some dimensions of the latent space capture specific morphologies (e.g., shmoo, hypha and pseudohypha on the left end of the first V1 dimension), there is a ubiquitous imperfect separation between all of the morphologies. This suggests that almost all morphologies have instances that are difficult to differentiate from one another. Yeast white cells span almost the entire first latent dimension, overlapping in some regions heavily with gray-like and opaque. Several technical artifacts including light intensity drive the scatterplot

especially in the first V1 dimension. The second V2 dimension primarily captures differences in the size and texture of the cells.

Conclusions. Variational autoencoders (VAEs) provide a convenient tool for modelling the diversity of cells caused by natural cellular programs, morphologies and technical artifacts. We observe a nearly unbroken, continuous distribution of points in our two-dimensional embedding, suggesting that the underlying space of *C. albicans* morphologies are also continuous and overlapping, as perhaps expected. We certainly cannot rule out the possibility that a more advanced architecture for the VAE would better separate the morphologies. However we stress that this specific VAE easily separates the vast majority of points in other deep learning sets including MNIST[4] and Omniglot[5], suggesting the *C. albicans* morphology is at least as difficult as these well-studied learning challenges.

Supplemental Figure 1. Description of the cumulative curriculum learning set. Panel **A** depicts the frequency of the different classes across the six grades in both training and validation. An image is part of all subsequent grades once it appears for the first time. Images are included in the training and validation sets at a ratio of 7:3. Panel **B** provides histograms of the number of objects (cells, junctions, unknown, artifacts) per image across all six grades. The maximum number of objects in any image was 97, although we note that several test set images exceeded this bound (not shown here).

Supplemental Figure 2. The curves for the three types of FCOS loss across the six grades for the chosen Candescence classifier (τ=0.25) from Table 2. Although the number of iterations (x-axis) varies across the grades because of differences in the size of the learning set for each grade, in all cases this translates to a total of 5,000 epochs. From this, an epoch number of 600 to 1,000 was chosen, as it appears that convergence has been reached after one-fifth of the

epochs. It is well-established that the center-ness loss converges to ~0.57. All other losses are negligibly above 0.

Supplemental Figure 3. A compendium of all false positive object predictions (hallucinations) produced by Candescence for white, opaque and gray classes. As in Figure 3(i), X indicates that we truly are not able to see an object (a true hallucination) and M indicates that Candescence was indeed correct to predict a bounding box at that location (missed by the human annotators) but its subsequent classification disagrees with our criteria. Subimages lacking an annotation indicate that Candescence correctly identified and classified the object, which represents a human error.

Supplemental Figure 4. Analogous to Supplemental Figure 3 but for the shmoo, hypha and pseudohypha, artifact and unknown classes.

Supplemental Figure 5. Exploration of the performance of Candescence on the test set. Each panel refers to one of ~1,000 test set images with the UID available in Supplemental Table 1.

Supplemental Figure 6. Continued exploration of the performance of Candescence on the test set. Both panels refer to one of ~1,000 test set images with the UID available in Supplemental Table 1.

Supplemental Figure 7 sketches the general design of our Generative Adversarial Network (GAN) trained on subimages. This design remains unchanged from the original presentation in Liu et al. (2021), although hyperparameters were fit using Varasana.

Supplemental Figure 8 depicts a scatterplot of the two dimensional latent space of the VAE on the training and validation datasets. The junction, hyphae, pseudohyphae, and unknown class have been removed from the training and visualization of the VAE. The V1 dimension of the

VAE is strongly associated with the light intensity of the image. The vertical V2 dimension captures other variability primarily related to size and texture of the cells.

Supplemental Table 1. The Varasana learning set. UID is a distinct integer for each file. Type corresponds to a single colony that was photographed under the microscope and repetition refers to individual images taken of that colony. Gene target 1-3 describe the specific genetic modifications. ON under the Time column indicates overnight for 24 hours total. Columns S-AM provide the (manually assigned) number of cells per class per image across the training and validation datasets.

References

1. Kingma DP and Welling M. Auto-Encoding Variational Bayes. ArXiv13126114 Cs Stat 2013.
2. Chollet F and Allaire J. R Interface to Keras. GitHub; 2017.
3. Higgins I, Chang L, Langston V, et al. Unsupervised Deep Learning Identifies Semantic Disentanglement in Single Inferotemporal Neurons. ArXiv200614304 Q-Bio 2020.
4. LeCun Y. The Mnist Database of Handwritten Digits. 1989. Available from: http://yann. lecun. com/exdb/mnist/.
5. Lake BM, Salakhutdinov R and Tenenbaum JB. Human-Level Concept Learning through Probabilistic Program Induction. Science 2015;350(6266):1332–1338; doi: 10.1126/science.aab3050.
